# Potential effects of heat waves on the population dynamics of the dengue mosquito *Aedes albopictus*

**DOI:** 10.1371/journal.pntd.0007528

**Published:** 2019-07-05

**Authors:** Pengfei Jia, Lu Liang, Xiaoyue Tan, Jin Chen, Xiang Chen

**Affiliations:** 1 State Key Laboratory of Earth Surface Processes and Resource Ecology, Beijing Normal University, Beijing, China; 2 China Academy of Urban Planning and Design, Beijing, China; 3 Department of Geography and the Environment, University of North Texas, Union Circle, Denton, Texas, United States of America; 4 Department of Geography, University of Connecticut, Storrs, Connecticut, United States of America; London School of Hygiene & Tropical Medicine, UNITED KINGDOM

## Abstract

Extreme weather events affect the development and survival of disease pathogens and vectors. Our aim was to investigate the potential effects of heat waves on the population dynamics of Asian tiger mosquito (*Aedes albopictus*), which is a major vector of dengue and Zika viruses. We modeled the population abundance of blood-fed mosquito adults based on a mechanistic population model of *Ae*. *albopictus* with the consideration of diapause. Using simulated heat wave events derived from a 35-year historical dataset, we assessed how the mosquito population responded to different heat wave characteristics, including the onset day, duration, and the average temperature. Two important observations are made: (1) a heat wave event facilitates the population growth in the early development phase but tends to have an overall inhibitive effect; and (2) two primary factors affecting the development are the unusual onset time of a heat wave and a relatively high temperature over an extended period. We also performed a sensitivity analysis using different heat wave definitions, justifying the robustness of the findings. The study suggests that particular attention should be paid to future heat wave events with an abnormal onset time or a lasting high temperature in order to develop effective strategies to prevent and control *Ae*. *albopictus-*borne diseases.

## Author summary

Understanding the population dynamics of Asian Tiger mosquito (*Ae*. *albopictus*)–the most prevalent vector of global epidemics including West Nile virus, dengue fever, Zika–could shed lights on improving the understanding of vector transmission as well as developing effective disease control strategies. It is widely acknowledged that the life cycle of *Ae*. *albopictus* is firmly regulated by meteorological factors in a non-linear way and is sensitive to climate change. Our study extends the understanding about how extreme heat events manipulate the mosquito population abundance. We adopted an existing mechanistic population model of *Ae*. *albopictus*, combined with a rich set of simulated heat wave events derived from a 35-year historical dataset, to quantify the mosquito’s responses to different heat wave characteristics. We found that an abnormal onset time and a lasting high temperature play the most important role in affecting the mosquito population dynamics. We also performed a sensitive analysis by changing the definition of the heat wave, justifying the rigor of the conclusion. This research provides implications for developing public health intervention strategies: to control dengue fever, Zika, as well as other far-reaching mosquito-borne epidemics, priority should be given to heat wave events with an abnormal onset time or a lasting high temperature.

## Introduction

Originated from Southeast Asia, Asian tiger mosquito (*Aedes albopictus*) is the most prevalent vector in all continents except the Antarctica [1, 2, 3, 4, 5]. The pathogens it transmits pose a severe threat to human health by global epidemics, including dengue and Zika arboviruses. For instance, the dengue incidence has increased six-fold from 1990 to 2013, with cases more than doubled every decade [6]. This historical evidence suggests a crucial need to develop effective disease control and intervention strategies in order to minimize the risk of epidemic spread and infection [7, 8]. Meanwhile, the Zika virus, being detrimental to children born with microcephaly and neurological disorders, has spread from Brazil to twenty-six other countries or territories in the Americas within one year [1]. Despite the increasing infections and rapid spread of these arboviruses, no effective antiviral treatment exists. Thus, controlling the development of mosquito vectors becomes a viable option for curbing the disease transmission, especially in regions with limited public health resources [9].

The life cycle and transmission of most infectious agents are inextricably linked to climate [10]. *Ae*. *albopictus* is a small-bodied ectotherm; its population abundance and dynamics are firmly regulated by meteorological factors [11] and are sensitive to climate change [5], [12]. Temperature influences many aspects of *Ae*. *albopictus*’ life cycle in a non-linear fashion [13, 14, 15]. Lukewarm temperature fosters the development of mosquito at the stages of egg incubation [13], larval pupation [14], and pupal eclosion [15]; and it shortens the extrinsic incubation period, eventually expediting the transmission cycle and adult production [15]. However, when temperature exceeds a certain threshold, the effects on the mosquito development become contrastingly different and even detrimental [15]. It was tested that the duration of the gonotrophic cycle or the oviposition extended and the number of laid eggs decreased when the temperature rose above 35.0°C [16]. Findings from forecasting models also proved that the mosquito population tended to decrease in certain tropical regions under extremely hot weather [17].

The mechanism leading to the population dynamics of *Ae*. *albopictus* has yet to be elucidated [14, 15, 16, 18]. An obstacle to the identification is the uncertainty of climatic conditions, such as the onset, peak, and duration of extreme weather events, which are globally heterogeneous and regionally specific. Furthermore, seeking the theoretic pathway to the mosquito development is becoming more challenging, since the global climate manifests a higher degree of oscillation [19, 20, 21]. A coupled global climate model predicts that heat waves, as common extreme weather events, will become more frequent and longer-lasting in the second half of the 21^st^ century [22]. Despite few instances exploring the statistical links between heat waves and the mosquito ecology, the climate-driven mechanism has been poorly understood [23]. Specifically, little is known about how heat wave characterisitics (e.g., the onset day of a heat wave, the duration of a heat wave) affect the development. This existing knowledge gap obfuscates developing effective strategies to prevent and control mosquito-borne epidemics.

Many studies have employed controlled experiments to identify the response of *Ae*. *albopictus* to extremely high temperature [14, 15, 16, 18]. These studies, however, cannot capture the full range of parameters in the mosquito’s life stages, since the development process is relatively slow, complicated, and unrepeatable. Statistical methods (e.g., multivariate regression models) are able to establish the long-term association between environmental factors and population growth, but they are invariably focused on the aquatic stages (e.g., larvae) and are thus unable to characterize the growth parameters in the aerial stages (i.e., adults) and explain the intricacy of the transition between stages [24]. Most importantly, the few recorded heat wave events at data collection sites pose a considerable challenge to the model validation. To overcome the data issue, computer-based simulations of the weather processes offer an alternative solution [25]; however, very few existing studies are focused on the impact of extreme weather [23]. In addition, most simulation studies rely on statistical models while overlooking the intrinsic process of the development within the mosquito’s life-history stages.

The mechanistic population model, which establishes the multi-stage development of the mosquito by a series of differential equations, has become popular in the entomological research of mosquitoes [26, 27, 28]. Recently, Jia et al. [29] proposed a mechanistic population model that accounts for the diapause behavior, referring to the inactive state in which the mosquito is unable to hatch and ceases from the development in order to survive extreme environmental conditions (e.g., high temperature, extreme desiccation). This model, termed the mechanistic population model of *Ae*. *albopictus* with diapause (MPAD), has been further explored in this paper to identify the mechanistic associations between heat waves and the population abundance of *Ae*. *albopictus*. A 35-year historical heat wave dataset was employed to extract key climatic elements. Finally, a rich set of mathematical simulations were conducted to thoroughly investigate the important mechanisms responsible for the population dynamics of *Ae*. *albopictus* caused by heat waves.

## Materials and methods

### Study area

**Fig 1:**
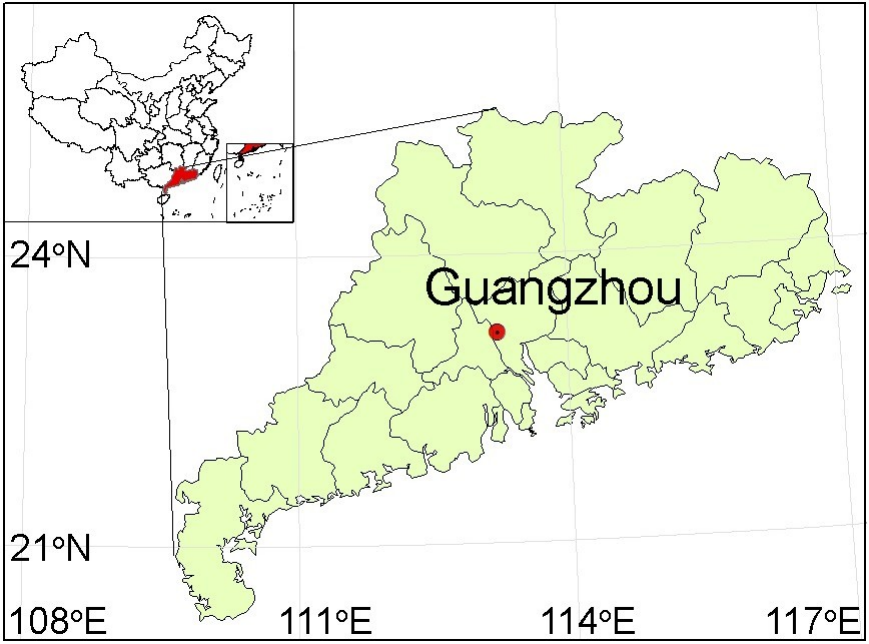
The study area: Guangzhou, China. The map was produced by ESRI ArcMap 10.4. The data used for the map was open access and was provided by ESRI ArcGIS Online.

Our study area is in Guangzhou (113.23°E, 23.17°N) (Fig 1)—the largest city of Southwest China with over 12.7 million population and a population density of 1,708 residents per km^2^ [30]. This mega-city has a distinct subtropical climate with an average annual temperature of 21.9°C and an annual rainfall ranging from 1,370 to 2,353 mm. The humid and warm climate is favorable for *Ae*. *albopictus* to survive and grow. In 2014, an unprecedented outbreak of dengue fever occurred in Guangzhou, causing 37,305 cases of infections [30]. This outbreak was attributed to the combined effects of the urban heat island and climate change, including more frequent and intense heat wave events [31].

### Mechanistic population model

The theoretical foundation of the study is the climate-driven and process-based MPAD model [29, 32]. The MPAD model formulates the continuous development of *Ae*. *albopictus* in a seven-stage process using a bottom-up approach, as shown in Eqs (1) through (7). These seven stages include eggs (*E*, including non-diapause *E*_0_ and diapause *E*_dia_, Eq (1)), larvae (*L*, Eq (2)), pupae (*P*, Eq (3)), emerging adults (*A*_em_, Eq (4)), blood-fed adults (*A*_b_, Eq (5)), gestating adults (*A*_g_, Eq (6)), and ovipositing adults (*A*_o_, Eq (7)). In each equation (representing one development stage), the variation of daily population abundance (marked in the prime notation) is determined by (1) the accumulated population from the last stage, (2) the mortality at the current stage, and (3) the population developing into the next stage. The life-history traits are driven by both climate-dependent parameters and climate-independent parameters. The climate-dependent parameters include daily mean temperature, daily accumulated precipitation, and daily photoperiod. These variables, derived from the experimental results [12, 16, 33, 34], are given in S1 Table and S2 Table. One highlight of the MPAD model is the consideration of diapause. Diapause-related parameters, as indicated by the subscript *dia* in Eqs (1) through (7), are defined to indicate whether the mosquito eggs are dormant or whether adults suspend the hatching activity under extreme conditions [35, 36].

The performance of the MPAD model was evaluated in our previous work by comparing against field *Ae*. *abopictus* container index (CI) in two Chinese cities: Guangzhou and Shanghai [29]. The coefficient of determination (*r*^2^) was 0.84 in Guangzhou and 0.90 in Shanghai, which showed a significant improvement over previous mechanistic population models. The better performance was attributed to the inclusion of diapause-related parameters and the modification of temperature-driven parameters. These adjustments are of critical importance in regions characterized by considerable seasonality (e.g., temperate zones), where the intra-annual dynamics of mosquito population only emerges with one peak.

$$\left\{ \begin{array}{c} {\dot{E}}_{0}=(1-z_{1})\beta A_{0}-(m_{E}+f_{E})E_{0}\\ {\dot{E}}_{\mathrm{dia}}=z_{1}\beta A_{0}-(m_{dia}+z_{2}f_{dia})E_{dia}\\ E=E_{0}+E_{dia} \end{array}\right.$$ (1)

$$\dot{L}=(f_{E}E_{0}+z_{2}f_{dia}E_{dia})-\left[m_{L}(1+\frac{L}{K_{L}})+f_{L}\right]L$$ (2)

$$\dot{P}=f_{L}L-(m_{P}+f_{P})P$$ (3)

$${\dot{A}}_{\mathrm{em}}=f_{P}\sigma e^{-\mu_{em}(1+\frac{P}{K_{P}})}P-(m_{A}+z_{dia}\gamma_{A_{em}})A_{em}$$ (4)

$${\dot{A}}_{b}=z_{dia}(\gamma_{A_{em}}A_{em}+\gamma_{A_{o}}A_{o})-(m_{A}+\mu_{r}+z_{dia}\gamma_{A_{b}})A_{b}$$ (5)

$${\dot{A}}_{r}=z_{dia}\gamma_{A_{b}}A_{b}-(m_{A}+f_{A_{g}})A_{g}$$ (6)

$${\dot{A}}_{0}=f_{A_{g}}A_{g}-(m_{A}+\mu_{r}+z_{dia}\gamma_{A_{o}})A_{o}$$ (7)

### Definition and characteristics of heat waves

The heat wave (HW) is an extended period of continuously hot weather, typically followed by a high level of humidity [22]. However, since local acclimatization and adaptation influence the impact of extreme heat, there is no globally accepted measure of heat waves [37]. A widely used strategy is to define heat wave locally using both intensity and duration indicators [38, 39]. Here, we first adopted one heat wave definition given by the China National Standard: a heat wave refers to an extreme weather event where the daily maximum temperature is greater than or equal to 35.0°C for at least three consecutive days (HW Definition I) [40].

To extract the historical heat wave events, we acquired all available daily temperature and precipitation measurement data in Guangzhou from the China Meteorological Data Sharing Service System, and generated a 35-year climate dataset spanning from 1980 to 2014 [41]. We also derived the photoperiod data from the National Oceanic and Atmospheric Administration [42]. Using the temperature dataset, we identified all heat waves in the study area.

**Fig 2:**
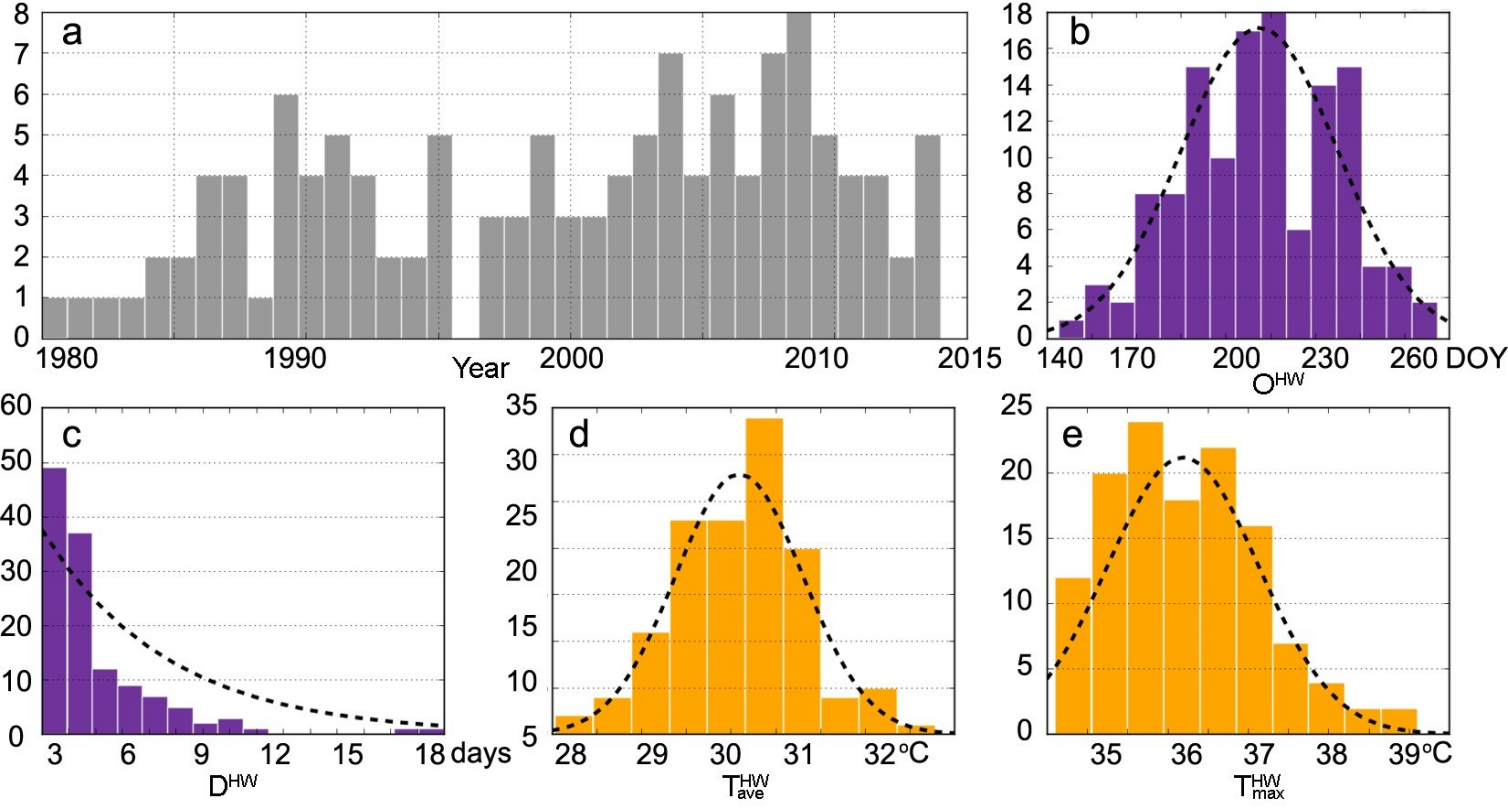
The frequency distributions of heat wave characteristics in Guangzhou, China (1980–2014). The Y-axes are the frequency (count), the X-axes are (a) year of occurrence, (b) onset day (DOY), (c) duration (days), (d) average daily mean temperature ($T_{ave}^{HW}$), (e) maximum of the daily maximum temperature ($T_{max}^{HW}$), respectively.

Heat wave events operate at both fast and slow rates with various degrees of severity. These processes can be characterized by the onset day (*O*^HW^, the first day in day of year [DOY] when a heat wave occurs), the duration (*D*^HW^, the period of consecutive heat wave days), and the average daily mean temperature ($T_{ave}^{HW}$). Their descriptive statistics are shown in Table 1. The frequency distributions (fit by trend curves) of their characteristics are summarized in Fig 2. The only year without an occurrence is 1996, after which an increased frequency can be identified (Fig 2A). The occurrences have a strong seasonality, where the most frequent DOYs range from mid-July to mid-August (Fig 2B). More than two-thirds of events (*n* = 86) have lasted three to four days (Fig 2C). In addition, the peak of $T_{ave}^{HW}$ ranges from 29.7–30.5°C (Fig 2D) and the peak of the maximum of the daily maximum temperature ($T_{max}^{HW}$) is around 35.5–36.8°C (Fig 2E).

**Table 1:** Descriptive statistics of heat wave characteristics based on HW Definition I (*n* = 127, 1980–2014).

	*O*^HW^ (DOY)	*D*^HW^ (days)	$T_{ave}^{HW}({}^{\circ}C)$
**Mean**	211	4.7	30.1
**S.D.**	26	2.5	0.71
**Min.**	144	3	28.0
**1**^**st**^ **quartile (Q1)**	193	3	29.7
**2**^**nd**^ **quartile (Q2)**	211	4	30.2
**3rd quartile (Q3)**	232	5	30.5
**Max.**	271	18	32.3

As the heat wave is a complex extreme weather event, the estimates of the recurrence probabilities of heat waves are used as the proxy for the temporality of their occurrences [43], which are calculated from the probability distributions (*Pdf*) of *O*^HW^,*D*^HW^, and $T_{ave}^{HW}$, as given by Eqs (8) through (10).

$$pdf(O^{HW})=exp\left\{-{\left(\frac{O^{HW}-211}{26}\right)}^{2}\right\}$$ (8)

$$pdf(D^{HW})=4.7e^{-4.7(D^{HW}-3)}+3,D^{HW}\geq 3$$ (9)

$$pdf(T_{ave}^{HW})=exp\left\{-{\left(\frac{T_{ave}^{HW}-30.1}{0.71}\right)}^{2}\right\}$$ (10)

### Creating synthetic temperature series

**Fig 3:**
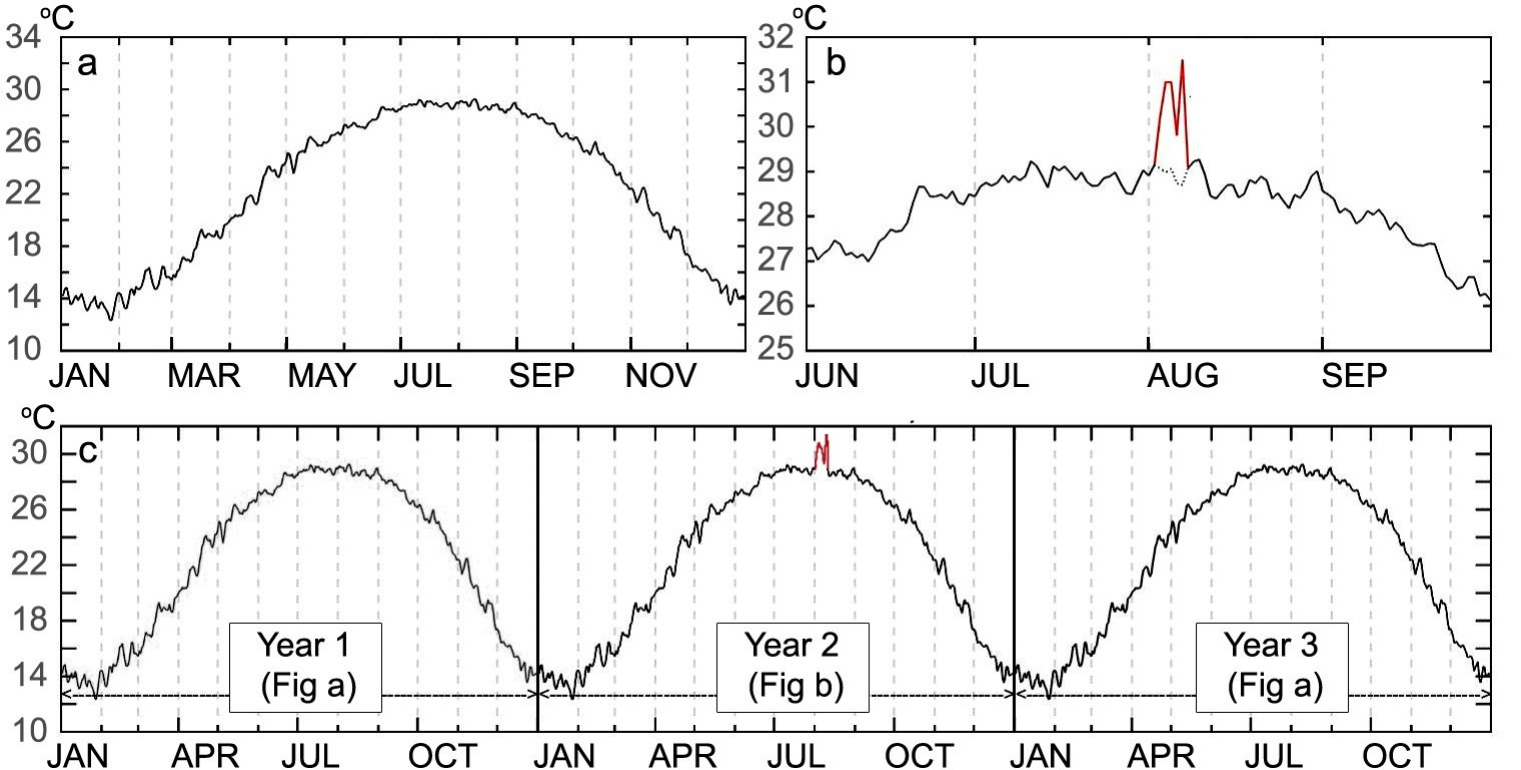
(a) 35-year averaged annual daily mean temperature series (*T*); (b) simulated heat wave temperature series (*T’*); (c) extrapolated 3-year temperature series, where Year 1 and Year 3 are derived from *T* and Year 2 is derived from *T’*.

In order to evaluate the effects of heat waves on population abundance, one assumption is to make the non-heat wave conditions constant across the period of observation without inter-annual variability. Thus, we calculated the averaged annual daily mean temperature (*T*) over 35 years using Eq (11), as shown in Fig 3A. We also derived the time series of two other key climatic variables required by the MPAD model: the daily accumulated precipitation (*P*) and the photoperiod (*PP*) (Eq 11). The temperature series *T* was then replaced by the temperature of a heat wave event (*T*^HW^, the red line in Fig 3B) during the heat wave DOYs (Eq 12). This new synthetic temperature series was labeled as *T*^*’*^ (Fig 3B). A total of 127 such temperature series *T’* were generated. In addition, we also found that using a single year is insufficient to identify the climate-driven mechanism, as the result is largely dependent on the conditions in Year 1 [27, 28, 29]. Thus, we extrapolated the 3-year temperature curve by placing *T’* in Year 2 (Fig 3C). We then designed experiments to test the effect of the 3-year temperature curve on the population abundance in Year 2 and Year 3.

$$X_{j}=\frac{1}{35}\sum_{i=1}^{35}X_{j}^{i}$$ (11)

*i* = 1…35 (year)

*j* = 1…365 (day)

*X* = *T*, *P*, *PP*

$$T^{\prime }=\left\{ \begin{array}{cc} T^{HW} & O^{HW}\leq DOYs\leq O^{HW}+D^{HW}\\ T & other\;DOYs \end{array}\right.$$ (12)

### Experimental design

The stage of blood-fed adults is of critical importance in the disease ecology. During this period, the mosquito becomes an active transmission vector of disease pathogens [44]. For this reason, we used the daily population abundance of the blood-fed adults to examine the heat wave effects.

Here we compared the population dynamics between two groups of blood-fed adults: the control group (*A*) under the non-heat wave scenario (*T*) and the test group (*A*^HW^) under the heat wave scenario (*T*^*’*^). After deriving the daily population abundance of *A* and *A*^HW^ by the MPAD model, we calculated the relative difference in population (*R*(*j*)), as shown in Eq (13). We then derived the duration of consecutive days (*RD*) when this relative difference exceeds 10% as a proxy for the heat wave effect, as shown in Eq (14).

$$R(j)=\frac{\left|A^{HW}(j)-A(j)\right|}{A(j)},j=1\ldots 365$$ (13)

$$RD={|t}_{E}-t_{B}|$$ (14)

where *t*_B_ denotes the first day when *R*(*j*) exceeds 10% and *t*_E_ denotes the last day when *R*(*j*) exceeds 10%.

Then we tested the effect of each heat wave characteristic. Specifically, the proposed indicator *RD* is treated as a function of three heat wave variables (*O*^HW^,*D*^HW^, and $T_{ave}^{HW}$), as shown in Eq (15).

$$RD∼f(O^{HW},D^{HW},T_{ave}^{HW})$$ (15)

To test the contribution of each climatic factor, we designed three groups of sensitivity analysis. In each group, only one factor was treated as a test variable while the two other factors were held constant as controlled variables, as shown in Table 2. For example, in the first group ({*RD*}~*O*^HW^), the value of *O*^HW^ was randomly drawn from its probability distribution (Eq (8)) for 1,000 times, while the two other factors *D*^HW^ and $T_{ave}^{HW}$ were selected as the combinations of their first, second, and third quartiles (the values were drawn from Table 1). This group of simulation generated a total of 9,000 runs.

**Table 2:** Experimental design to test the effect of each heat wave characteristic.

Group	Test variable	Controlled variable	
***RD*~*O***^**HW**^	*O*^HW^: 1000 random draws from Eq (8)	*D*^HW^ = 3 (Q1)	$T_{ave}^{HW}=Q1,Q2,\;or\;Q3$
		*D*^HW^ = 4(Q2)	$T_{ave}^{HW}=Q1,Q2,\;or\;Q3$
		*D*^HW^ = 5 (Q3)	$T_{ave}^{HW}=Q1,Q2,\;or\;Q3$
***RD*~*D***^**HW**^	*D*^HW^: 1000 random draws from Eq (9)	$T_{ave}^{HW}=29.7$ (Q1)	*O*^HW^ = Q1,Q2, or Q3
		$T_{ave}^{HW}=30.2$ (Q2)	*O*^HW^ = Q1,Q2, or Q3
		$T_{ave}^{HW}=30.5$ (Q3)	*O*^HW^ = Q1,Q2, or Q3
$RD\sim T_{ave}^{HW}$	$T_{ave}^{HW}$: 1000 random draws from Eq (10)	*O*^HW^ = 193 (Q1)	*D*^HW^ = Q1,Q2, or Q3
		*O*^HW^ = 211 (Q2)	*D*^HW^ = Q1,Q2, or Q3
		*O*^HW^ = 232 (Q3)	*D*^HW^ = Q1,Q2, or Q3

## Results

### Heat wave effects on mosquito population dynamics

**Fig 4:**
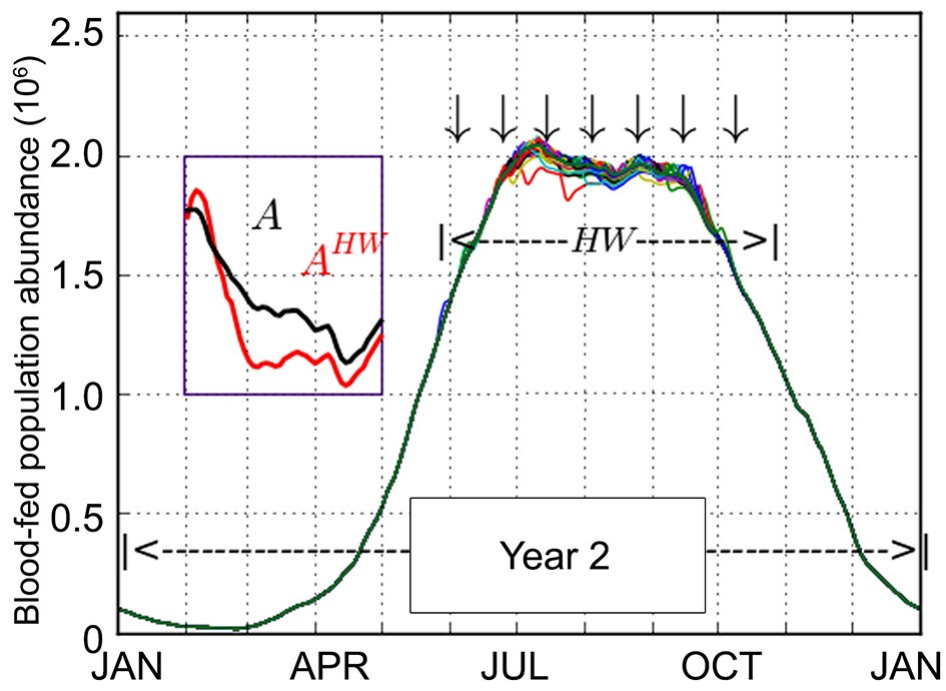
Overlay of simulated blood-fed adult populations *A*^HW^ derived from 127 heat wave temperature series *T’*. The inset shows the comparison between one selected *A*^HW^ (red curve, under one heat wave scenario *T’*) and *A* (black curve, under the non-heat wave scenario *T*).

The given heat wave definition generated a total of 127 synthetic heat wave temperature series *T’*. These series of *T’* served as the input into the MPAD model, further generating 127 daily blood-fed adult population abundance curve *A*^HW^ as the outcome. Comparatively, the population abundance *A* under the non-heat wave scenario *T* was also derived. The overlay of simulated heat wave population curves *A*^HW^ is shown in Fig 4, which reveals that the historical heat waves only occurred briefly from early summer into early autumn (DOYs∈[144,271],*D*^HW^∈[3,18]). Their effects on the population abundance were also limited to the time period when heat waves stroke and would not carry over to winter or the next year. In addition, the heat wave occurrences mostly suppressed the mosquito development rather than promoted it, as demonstrated by comparing *A* and one selected *A*^HW^ (Fig 4 inset).

### Effects of heat wave characteristics

**Fig 5:**
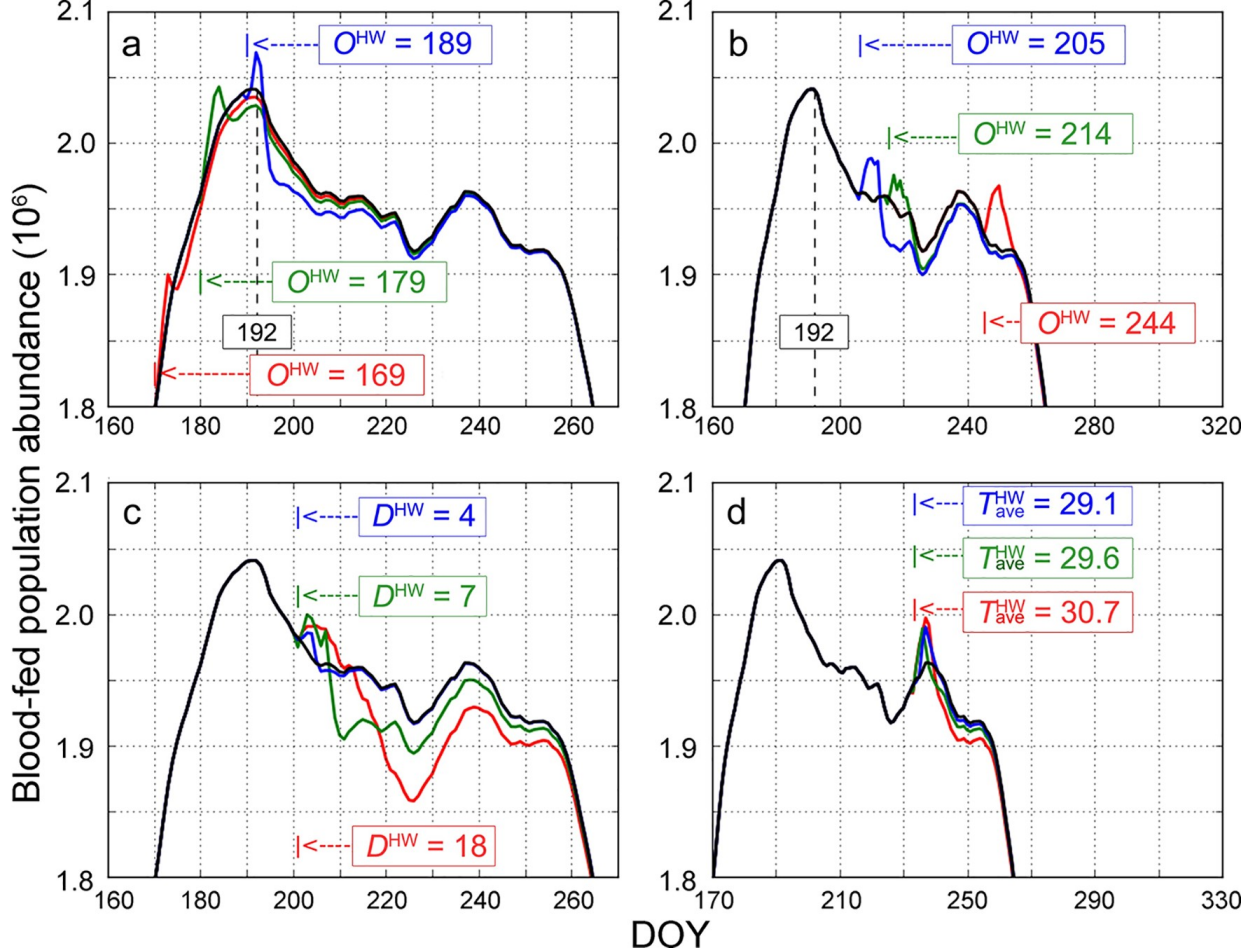
Simulated population curves by the MPAD model under different heat wave scenarios: (a) different *O*^HW^ (before DOY 192), (b) different *O*^HW^ (after DOY 192), (c) different *D*^HW^, and (d) different $T_{ave}^{HW}$. The black curve represents the population abundance under the non-heat wave scenario, where the peak is identified at DOY 192.

We further examined how the population dynamics responded to the variation of individual heat wave characteristics, including *O*^HW^(Fig 5A and 5B), *D*^HW^(Fig 5C), and $T_{ave}^{HW}$ (Fig 5D). For each test variable, we held the other two variables constant and included three specific cases for discussion. In addition, we generated the population abundance under the non-heat wave scenario *T* (black curve in Fig 5) and derived its peak at DOY 192.

Fig 5A shows the examples of three heat waves with different onset days (i.e., *O*^HW^ = 169, 179, and 189). These scenarios, with an onset day earlier than DOY 192, generated population curves similar to that under the non-heat wave scenario. The early onset of heat wave slightly advances the emergence of the population peak but has no cascading effect on the late stage development (DOY > 225). However, when heat waves occur after DOY 192 (i.e., *O*^HW^ = 205, 214, and 244), the population curves largely shift, where a greater level of variation is observed (Fig 5B). Fig 5C shows three heat waves with different durations (i.e., *D*^HW^ = 4, 7, and 18), which demonstrates that the longer the event lasts, the greater extent it suppresses the population growth. Lastly, Fig 5D shows three scenarios under different temperature conditions (i.e., $T_{ave}^{HW}$ = 29.1, 29.6, and 30.7), where the resulting effects on the population abundance are not significant. One noticeable pattern in all of these scenarios is that the population grows when the heat wave strikes but plummets after a short period. Several factors may contribute to this phenomenon. Environmentally, long-lasting heat waves can dry up shallow bodies of water and subsequently deprive mosquitos of breeding grounds. Physiologically, heat waves can also cause most mosquito species to spawn at once and then dry in unison when weather becomes extreme. Several genes of heat shock protein—known to overcome high temperature stress—tend to show downregulation in larvae when subject to thermal stress at 39°C [45].

**Fig 6:**
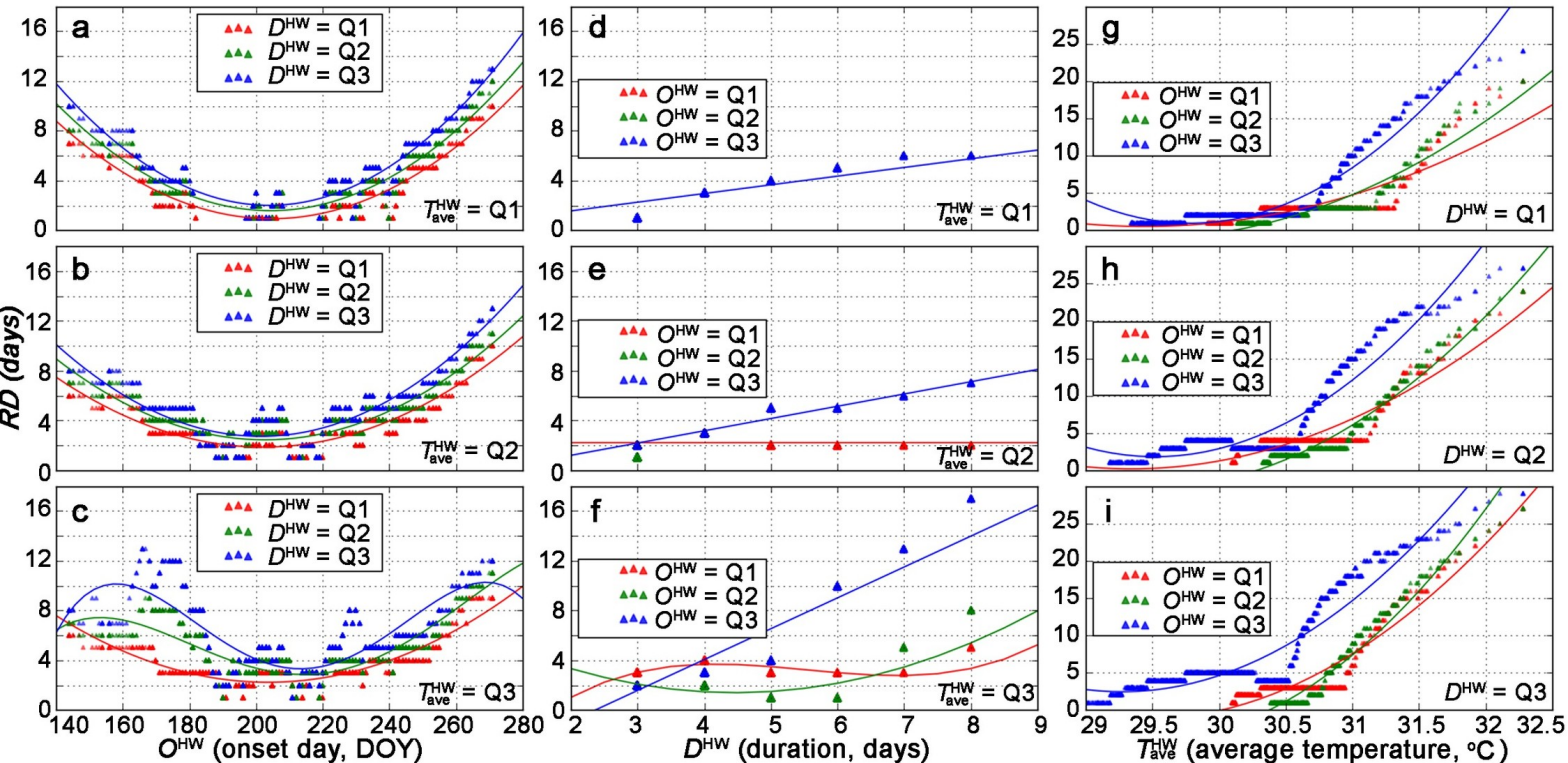
The relationships between *RD* and heat wave characteristics: (a-c) *O*^HW^, (d-f) *D*^HW^, and (g-i) $T_{ave}^{HW}$. Controlled variables are chosen as their first (Q1), second (Q2), and third quartile (Q3).

Besides the visual assessment, we further quantified the effects via statistical regressions based on the experimental design in Table 2. Specifically, we used *RD*—consecutive days when the relative difference in the population abundance exceeds 10%—as a population index representing the heat wave effects. Fig 6 shows the associations between *RD* and *O*^HW^,*D*^HW^, $T_{ave}^{HW}$. In Fig 6A–6C, the relationship between *RD* and *O*^HW^ generally follows a quadratic form (average *r*^2^ is around 0.90) with the trough appearing in late July (DOY 203–204). We noticed that in Fig 6C, in addition to the quadratic curve, two peaks emerge in early June (DOY 160) and late September (DOY 265) when both *D*^HW^ and $T_{ave}^{HW}$ are at their third quantiles (i.e, blue curve in Fig 6C). In Fig 6D–6F, a significant linear correlation is observed between *RD* and *D*^HW^ only with a large *O*^HW^(i.e., late heat wave onset, blue lines in Fig 6D–6F). In Fig 6G–6I, *RD* and $T_{ave}^{HW}$ have a piecewise association, which is relatively flat before $T_{ave}^{HW}$ = 30.5 and follows a linear pattern afterwards. The full list of the mathematical relationships are included in S3 Table.

### Sensitivity analysis of heat wave definitions

The definition of a heat wave event is regionally specific [39]. Since there is a lack of consensus about the heat wave definition, we would like to examine if our results are robust when a different definition applies. To test the sensitivity of the MPAD model, we adopted two other heat wave definitions that have been previously employed in Guangzhou [46, 47]. The second definition is less restrict: a heat wave is defined as ≥ 2 consecutive days with the daily mean temperature at or above the 95^th^ percentile of the year (HW Definition II) [46]. The last definition is a stricter criterion: a heat wave is defined as ≥ 7 consecutive heat days with the daily mean temperature at or above the 95^th^ percentile (HW Definition III) [47].

Based on the same experimental design, we extracted the historical heat waves according to each new definition. Their descriptive statistics are shown in S4 Table. Then, we simulated the mosquito population *A*^HW^ under each new heat wave definition and tested the relationship between *RD* and the three heat wave variables *O*^HW^,*D*^HW^, and $T_{ave}^{HW}$ following the simulation design in Table 2. The results are shown in S1 Fig (for HW Definition II) and S2 Fig (for HW Definition III). A total of 489 heat waves were extracted by using HW Definition II. It can be observed from the results that the correlation patterns are in consistent with HW Definition I. However, when HW Definition III was employed, only 12 heat waves were extracted. With the few identified events, we were unable to establish a significant correlation pattern. It is thus demonstrated that our simulation results are robust, when a sufficient number of observations can be generated using a new definition.

## Discussion

Although the evidence of heat wave effects on human health has grown significantly, the outcomes on important vectors such as mosquitoes remain unclear. Utilizing a mechanistic population model, we simulated the responses of *Ae*. *albopictus* to heat waves under various scenarios. The mechanistic model is advantageous in connecting the climatic parameters to the mosquito population abundance, which allows the mathematical interpretation of the multi-stage development behavior in the mosquito’s life cycle [26, 27, 28]. Although part of this work was initially inspired by laboratory experiments, controlled lab environments are constrained by the subject quantities and cannot fully capture the adult stage of the mosquito. Considering these limitations, we designed the MPAD model to simulate the life-history traits of the mosquito.

As our analyses illustrate, the historical heat wave events primarily have negative effects on the mosquito abundance. It is observed that a heat wave event could facilitate the population growth in the early development phase but tends to have an overall inhibitive effect (Fig 5). This conclusion was in line with previous knowledge: the preferable temperature range for the *Aedes* mosquito to develop is 15–30°C and the temperature of natural eradication is above 35°C [48]. Although the mosquito development can be promoted by a lukewarm temperature, it can be greatly curtailed when the temperature rises to a lethal threshold, which is not unusual in summer. Thus, when a heat wave occurs and persists (i.e., daily maximum temperature ≥ 35°C for at least three days), the mosquito development will be greatly inhibited.

Our research also provides insights into the heat wave-mosquito relations according to the distinct characteristics of the extreme weather, which, to the best of our knowledge, is among the first to be investigated. Most previous studies solely relied on the degree of temperature to identify the thermal reaction norm of mosquitoes [15, 48]. Supported by the systematic simulation, we found that heat waves with distinct characteristics, including the onset day, duration, and average temperature, can manipulate the mosquito dynamics during the heat wave period.

The onset time *O*^HW^ plays a complex role in manipulating the population dynamics. The quadratic relationship between the population index *RD* and *O*^HW^ (Fig 6A–6C) further confirms that the change of the mosquito population is less influenced by a heat wave, if the event emerges in late July. In Guangzhou, July is the month with the highest daily mean temperature; and for this reason, the mosquito could naturally adapt to the extreme weather. However, if the heat wave strikes relatively early or late, the mosquito population may not well adapt to the changing seasonality; thus, their development will exhibit different degrees of abnormity.

The effects of duration *D*^HW^ and the daily mean temperature $T_{ave}^{HW}$ are more complex. It can be observed that the relationships between the population index *RD* and *D*^HW^ are evident only when $T_{ave}^{HW}$ is high (Fig 6F). Also, the relationships between the population index *RD* and $T_{ave}^{HW}$ are in the forms of piecewise functions: only when $T_{ave}^{HW}$ rises above a threshold (i.e., 30.5°C in our simulation results) can the reduction in population becomes noticeable (Fig 6G–6I). These observations signify that a heat wave with a modest high temperature will not affect the development, no matter how long it lasts. The mosquito population will be significantly reduced only if the temperature rises beyond a certain threshold.

So far, we have illustrated the capacity and strength of the MPAD model in simulating the population dynamics of *Ae*. *albopictus*. Our study is also the first to assess the climatic influence on the mosquito using different heat wave definitions. Our conclusions are relatively robust when a different heat wave definition applies. Because of the nature of simulation models, we still recommend that regional specific investigations are needed to better define and articulate heat wave events, as the mosquito may evolve and adapt to a different level of climatic conditions [49].

Several limitations in this study should be mentioned. First, only static multivariate analysis was employed to investigate the regression between the population dynamics and heat wave characteristics, while ignoring the time lag effect. Many previous studies have identified the crucial role of time lag effect in dictating dengue incidence rates [49, 50, 51]. Thus, future research could consider employing more advanced statistical tools, such as the lagged-time Poisson regression analysis [52], to refine the experimental design. Second, this study has an overarching focus on the heat wave effects, whereas other extreme weather types (e.g., rainstorms) that could be equally important in altering the population abundance [53, 54], are overlooked. Future attempts should be made to investigate the climate-mosquito mechanisms under different extreme weather scenarios.

### Conclusions

There is much epidemiological evidence demonstrating how climate variations and trends affect human health outcomes [55, 56, 57]. Despite the many explorations on the disease pathogens, the complicated interplay between heat waves and *Ae*. *albopictus* remains unclear. This paper explores the variability of *Ae*. *albopictus* responding to heat waves events using a 35-year historical climate dataset via mathematical modeling and a simulation design. Our simulation results reveal that the unusual onset of a heat wave and a relatively high temperature over an extended period are the two primary factors inhibiting the population development. As the frequency and severity of heat waves are likely to increase in the future [22], this study provides insights into assessing the potential effects on the mosquito introduced by the global climate. Understanding this climate-driven mechanism is crucial to developing effective strategies to prevent and control dengue fever, Zika, as well as other far-reaching mosquito-borne epidemics.

## Supporting information

S1 Table MPAD model parameters, sourced from Jia et al. [24].
(DOCX)

S2 Table Formulation of climate-dependent parameters in the MPAD model, sourced from Jia et al. [24].
(DOCX)

S3 Table Mathematical relationships showing the effect of individual heat wave characteristics (under HW Definition I) on the population dynamics of *Aedes albopictus*.
(DOCX)

S4 Table Descriptive statistics of heat wave characteristics based on HW Definition II and Definition III.
(DOCX)

S1 FigThe relationships between *RD* and heat wave characteristics: (a-c) *O*^HW^, (d-f) *D*^HW^, and (g-i) $T_{ave}^{HW}$ based on HW Definition II. Controlled variables are chosen as their first (Q1), second (Q2), and third quartile (Q3).(TIF)

S2 FigThe relationships between *RD* and heat wave characteristics: (a-c) *O*^HW^, (d-f) *D*^HW^, and (g-i) $T_{ave}^{HW}$ based on HW Definition III. Controlled variables are chosen as their first (Q1), second (Q2), and third quartile (Q3).(TIF)
