# Supplementary material for: Potential effects of heat waves on the population dynamics of the dengue mosquito Aedes albopictus
Source: PLoS Negl Trop Dis. 2019 Jul 5;13(7):e0007528. doi: 10.1371/journal.pntd.0007528 (PMC6645582; doi:10.1371/journal.pntd.0007528)
Supplement: S1 Table — MPAD model parameters, sourced from Jia et al. [24]. (DOCX) [file pntd.0007528.s001.docx]

**S1 Table. MPAD model parameters, sourced from Jia et al. [24].**

| Climate-dependent parameters | | |
| --- | --- | --- |
| *f*_E_ | Non-diapause egg hatching rate | |
| *f*_dia_ | Diapause egg hatching rate | |
| *f*_L_ | Larva development rate | |
| *f*_P_ | Pupa development rate | |
| *m*_L_ | Larva mortality rate | |
| *m*_P_ | Pupa mortality rate | |
| *m*_A_ | Adult mortality rate | |
| *β* | Oviposition rate by each female | |
| *f*_Ag_ | Gestating adult development rate | |
| *k*_L_ | Environmental carrying capacity for larvae | |
| *k*_P_ | Environmental carrying capacity for pupae | |
| *z*_1_ | Binary function for diapause eggs oviposited | |
| *z_2_* | Binary function for diapause egg hatching | |
| *z*_dia_ | Binary function for adult activity during diapause period | |
|  | | |
| Climate-independent parameters | | |
| *m_E_* | | Non-diapause egg mortality rate |
| *m_dia_* | | Diapause egg mortality rate |
| *σ* | | Percentage of females at emerging adult stage |
| *μ_em_* | | Emerging adult mortality rate |
| *μ_r_* | | Adult mortality rate related to seeking behavior |
| *γ_Aem_* | | Emerging adult development rate |
| *γ_Ab_* | | Blood-fed adult development rate |
| *γ_Ao_* | | Ovipositing adult development rate |
