# Supplementary material for: Potential effects of heat waves on the population dynamics of the dengue mosquito Aedes albopictus
Source: PLoS Negl Trop Dis. 2019 Jul 5;13(7):e0007528. doi: 10.1371/journal.pntd.0007528 (PMC6645582; doi:10.1371/journal.pntd.0007528)
Supplement: S2 Table — Formulation of climate-dependent parameters in the MPAD model, sourced from Jia et al. [24]. (DOCX) [file pntd.0007528.s002.docx]

**S2 Table. Formulation of climate-dependent parameters in the MPAD model, sourced from Jia et al. [24].**

| Parameter | Equation |
| --- | --- |
| *f*_E_ | $f_{E}\left( T \right)=0.5070\exp\left[ -\left( \frac{T-30.85}{12.82} \right)^{2} \right]$ |
| *f*_dia_ | $f_{\mathrm{dia}}\left( T \right)=0.1*0.5070\exp\left[ -\left( \frac{T-30.85}{12.82} \right)^{2} \right]$ |
| *f*_L_ | $f_{L}\left( T \right)=0.1727\exp\left[ -\left( \frac{T-28.40}{10.20} \right)^{2} \right]$ |
| *f*_P_ | $f_{P}\left( T \right)=0.6020\exp\left[ -\left( \frac{T-34.29}{15.07} \right)^{2} \right]$ |
| *m*_L_ | $m_{L}\left( T \right)=min\left\{ 1,\frac{1}{\vert-0.1305T^{2}+3.868T+30.83\vert} \right\}$ |
| *m_­_*_p_ | $m_{P}\left( T \right)=min\left\{ 1,\frac{1}{\vert-0.1502T^{2}+5.057T+3.517\vert} \right\}$ |
| *m*_A_ | $m_{A}\left( T \right)=min\left\{ 1,\frac{1}{\vert-0.1921T^{2}+8.147T-22.98\vert} \right\}$ |
| *β* | $\beta\left( T \right)=max\left\{ 0,-0.0163T^{2}+1.2897T-15.837 \right\}$ |
| *f*_Ag_ | $f_{\mathrm{Ag}}\left( T \right)=max\left\{ 0,\frac{T-10}{77} \right\}$ |
| *k*_L_ | $k_{L}\left( PP_{norm} \right)=\kappa_{L}(1+PP_{norm})$ |
| *k*_P_ | $k_{P}\left( PP_{norm} \right)=\kappa_{P}(1+PP_{norm})$ |
| *z*_1_ | $z_{1}\left( T_{ave},SD_{ave} \right)=$  $\left\{ \begin{aligned} 1, T_{ave}\left( t \right)<21℃ and SD_{ave}\left( t \right)<13.5h, t_{eggBegin}<t<t_{diaBegin} \\ 0, otherwise \end{aligned} \right.$ |
| *z_2_* | $z_{2}\left( T_{ave},SD_{ave} \right)=$  $\left\{ \begin{aligned} 1, T_{ave}\left( t \right)>10.5℃ and SD_{ave}\left( t \right)>11.25h, t_{diaEnd}<t<t_{eggEnd} \\ 0, otherwise \end{aligned} \right.$ |
| *z*_dia_ | $z_{2}\left( T_{ave},SD_{ave} \right)=$  $\left\{ \begin{aligned} 1, T_{ave}\left( t \right)<9.5℃, t>t_{diaBegin} or t<t_{diaEnd} \\ 0, otherwise \end{aligned} \right.$ |

*Abbreviations*: *T* - daily mean temperature, *PP*_norm_ - rainfall (precipitation, normalized between 0 and 1) over a 2-week period, *T*_ave_ - 7-day averaged daily mean temperature, *SD*_ave_ - 7-day averaged daily sunlight hour (photoperiod), *t*_eggBegin_ - the emergence time of diapause eggs, *t*_eggEnd_ - the time when diapause eggs finish hatching, *t*_diaBegin_ - the onset of diapause period, *t*_diaEnd_ - the ending time of diapause period.
