# Supplementary material for: Potential effects of heat waves on the population dynamics of the dengue mosquito Aedes albopictus
Source: PLoS Negl Trop Dis. 2019 Jul 5;13(7):e0007528. doi: 10.1371/journal.pntd.0007528 (PMC6645582; doi:10.1371/journal.pntd.0007528)
Supplement: S3 Table — Mathematical relationships showing the effect of individual heat wave characteristics (under HW Definition I) on the population dynamics of Aedes albopictus. (DOCX) [file pntd.0007528.s003.docx]

**S3 Table. Mathematical relationships showing the effects of individual heat wave characteristics (under HW Definition I) on the population dynamics of *Aedes albopictus.***

|  | | | y = *RD* |
| --- | --- | --- | --- |
| ${\boldsymbol{x}\mathbf{=}\boldsymbol{O}}^{\mathbf{HW}}$ | $T_{\mathrm{ave}}^{\mathrm{HW}}=Q1$ | $D^{\mathrm{HW}}=Q1$ | *y* = 80 – 0.8*x* + 1.9e-3*x*^2^, *r^2^* = 0.88 |
|  |  | $D^{\mathrm{HW}}=Q2$ | *y* = 88 – 0.9*x* + 2.0e-3*x*^2^, *r^2^* = 0.89 |
|  |  | $D^{\mathrm{HW}}=Q3$ | *y* = 101 – 1.0*x* + 2.4e-3*x*^2^, *r^2^* = 0.92 |
|  | $T_{\mathrm{ave}}^{\mathrm{HW}}=Q2$ | $D^{\mathrm{HW}}=Q1$ | *y* = 61 – 0.6*x* + 1.5e-3*x*^2^, *r^2^* = 0.86 |
|  |  | $D^{\mathrm{HW}}=Q2$ | *y* = 71 – 0.7*x* + 1.6e-3*x*^2^, *r^2^* = 0.86 |
|  |  | $D^{\mathrm{HW}}=Q3$ | *y* = 82 – 0.8*x* + 2.0e-3*x*^2^, *r^2^* = 0.84 |
|  | $T_{\mathrm{ave}}^{\mathrm{HW}}=Q3$ | $D^{\mathrm{HW}}=Q1$ | *y* = 57 – 0.5*x* + 1.3e-3*x*^2^, *r^2^* = 0.83 |
|  |  | $D^{\mathrm{HW}}=Q2$ | *y* = – 514 + 10*x* – 7.6e-2*x*^2^ + 2.4e-4*x*^3^ – 2e-7*x*^4^, *r^2^* = 0.75 |
|  |  | $D^{\mathrm{HW}}=Q3$ | *y* = – 1302 + 26*x* – 1.9e-1*x*^2^ + 6.2e-4*x*^3^ – 7.3e-7*x*^4^, *r^2^* = 0.62 |
| ${\boldsymbol{x}\mathbf{=}\boldsymbol{D}}^{\mathbf{HW}}$ | $T_{\mathrm{ave}}^{\mathrm{HW}}=Q1$ | $O^{\mathrm{HW}}=Q1$ | N/A |
|  |  | $O^{\mathrm{HW}}=Q2$ | N/A |
|  |  | $O^{\mathrm{HW}}=Q3$ | *y* = –0.2 + 0.7*x*, *r^2^* = 0.88 |
|  | $T_{\mathrm{ave}}^{\mathrm{HW}}=Q2$ | $O^{\mathrm{HW}}=Q1$ | *y* = 2.2 |
|  |  | $O^{\mathrm{HW}}=Q2$ | N/A |
|  |  | $O^{\mathrm{HW}}=Q3$ | *y* = –0.8 + 1.0*x*, *r^2^* = 0.95 |
|  | $T_{\mathrm{ave}}^{\mathrm{HW}}=Q3$ | $O^{\mathrm{HW}}=Q1$ | *y* = –9.4 + 7.8*x* – 1.5*x* ^2^ + 0.1*x* ^3^, *r^2^* = 0.92 |
|  |  | $O^{\mathrm{HW}}=Q2$ | *y* = 7.7 – 2.8*x* + 0.3*x* ^2^, *r^2^* = 0.91 |
|  |  | $O^{\mathrm{HW}}=Q3$ | *y* = –5.9 + 2.5*x*, *r^2^* = 0.94 |
| ${\boldsymbol{x}\mathbf{=}\boldsymbol{T}}_{\mathbf{ave}}^{\mathbf{HW}}$ | $D^{\mathrm{HW}}=Q1$ | $O^{\mathrm{HW}}=Q1$ | *y* = 1513 – 103*x* + 1.7*x^2^*, *r^2^* = 0.70 |
|  |  | $O^{\mathrm{HW}}=Q2$ | *y* = 2061 – 140*x* + 2.4 *x^2^*, *r^2^* = 0.88 |
|  |  | $O^{\mathrm{HW}}=Q3$ | *y* = 4464 – 300*x* + 5.0 *x^2^*, *r^2^* = 0.93 |
|  | $D^{\mathrm{HW}}=Q2$ | $O^{\mathrm{HW}}=Q1$ | *y* = 2093 – 143*x* + 2.4*x^2^*, *r^2^* = 0.81 |
|  |  | $O^{\mathrm{HW}}=Q2$ | *y* = 3085 – 210*x* + 3.6*x^2^*, *r^2^* = 0.94 |
|  |  | $O^{\mathrm{HW}}=Q3$ | *y* = 4089 – 277*x* + 4.7*x^2^*, *r^2^* = 0.87 |
|  | $D^{\mathrm{HW}}=Q3$ | $O^{\mathrm{HW}}=Q1$ | *y* = 3077 – 209*x* + 3.6*x^2^*, *r^2^* = 0.88 |
|  |  | $O^{\mathrm{HW}}=Q2$ | *y* = 4154 – 282*x* + 4.8*x^2^*, *r^2^* = 0.95 |
|  |  | $O^{\mathrm{HW}}=Q3$ | *y* = 3497 – 239*x* + 4.1*x^2^*, *r^2^* = 0.87 |

*Abbreviations*: $O^{\mathrm{HW}}$ - the onset day in DOY when a heat wave occurs, $D^{\mathrm{HW}}$ - the duration in consecutive days a heat wave occurs, $T_{\mathrm{ave}}^{\mathrm{HW}}$ - the averaged daily mean temperature during a heat wave, *RD* - consecutive days when the relative difference in the population abundance exceeds 10%, as shown in Equation (14), Q1 - first quartile, Q2 - second quartile, Q3 - third quartile, and *r*^2^ - correlation coefficient of the regression.
