# Supplementary material for: Potential effects of heat waves on the population dynamics of the dengue mosquito Aedes albopictus
Source: PLoS Negl Trop Dis. 2019 Jul 5;13(7):e0007528. doi: 10.1371/journal.pntd.0007528 (PMC6645582; doi:10.1371/journal.pntd.0007528)
Supplement: S4 Table — Descriptive statistics of heat wave characteristics based on HW Definition II and Definition III. (DOCX) [file pntd.0007528.s004.docx]

**S4 Table. Descriptive statistics of heat wave characteristics based on HW Definition II and Definition III.**

|  | HW Definition II* (*n* = 489) | | | HW Definition III^**^ (*n* = 12) | | |
| --- | --- | --- | --- | --- | --- | --- |
|  | $\boldsymbol{O}^{\mathbf{HW}}$ (DOY) | $\boldsymbol{D}^{\mathbf{HW}}$ (days) | $\boldsymbol{T}_{\mathbf{ave}}^{\mathbf{HW}}$ (^o^C) | $\boldsymbol{O}^{\mathbf{HW}}$ (DOY) | $\boldsymbol{D}^{\mathbf{HW}}$ (days) | $\boldsymbol{T}_{\mathbf{ave}}^{\mathbf{HW}}$ (^o^C) |
| Mean | 205 | 3.4 | 30.0 | 202 | 8.3 | 30.8 |
| S.D. | 26 | 1.8 | 0.80 | 23 | 1.5 | 0.70 |
| Min. | 145 | 2 | 27.9 | 171 | 7 | 29.6 |
| 1^st^ quartile (Q1) | 188 | 2 | 29.6 | 182 | 7 | 30.4 |
| 2^nd^ quartile (Q2) | 206 | 3 | 30.1 | 207 | 8 | 30.6 |
| 3rd quartile (Q3) | 224 | 4 | 30.5 | 213 | 9 | 31.6 |
| Max. | 265 | 11 | 31.9 | 241 | 11 | 31.7 |

*HW Definition II is ≥2 consecutive days with the daily mean temperature at or above the 95th percentile of the year.

**HW Definition III is ≥ 7 consecutive heat days with the daily mean temperature at or above the 95th percentile of the year.
