# Supplementary figures and images for: Potential effects of heat waves on the population dynamics of the dengue mosquito Aedes albopictus
Source: PLoS Negl Trop Dis. 2019 Jul 5;13(7):e0007528. doi: 10.1371/journal.pntd.0007528 (PMC6645582; doi:10.1371/journal.pntd.0007528)

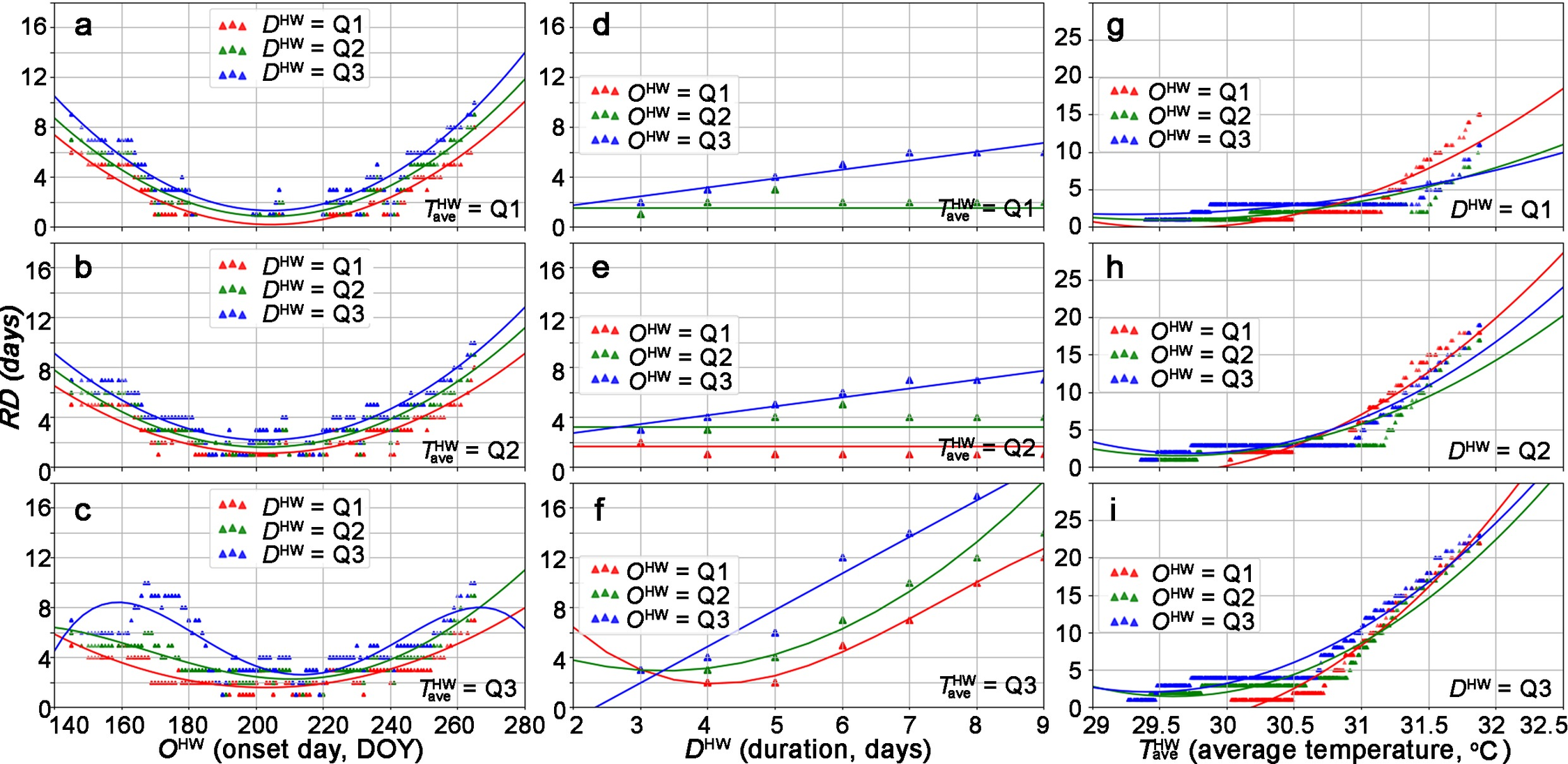

Supplement: S1 Fig — The relationships between RD and heat wave characteristics: (a-c) OHW, (d-f) DHW, and (g-i) TaveHW based on HW Definition II. Controlled variables are chosen as their first (Q1), second (Q2), and third quartile (Q3). (TIF) [file pntd.0007528.s005.tif]

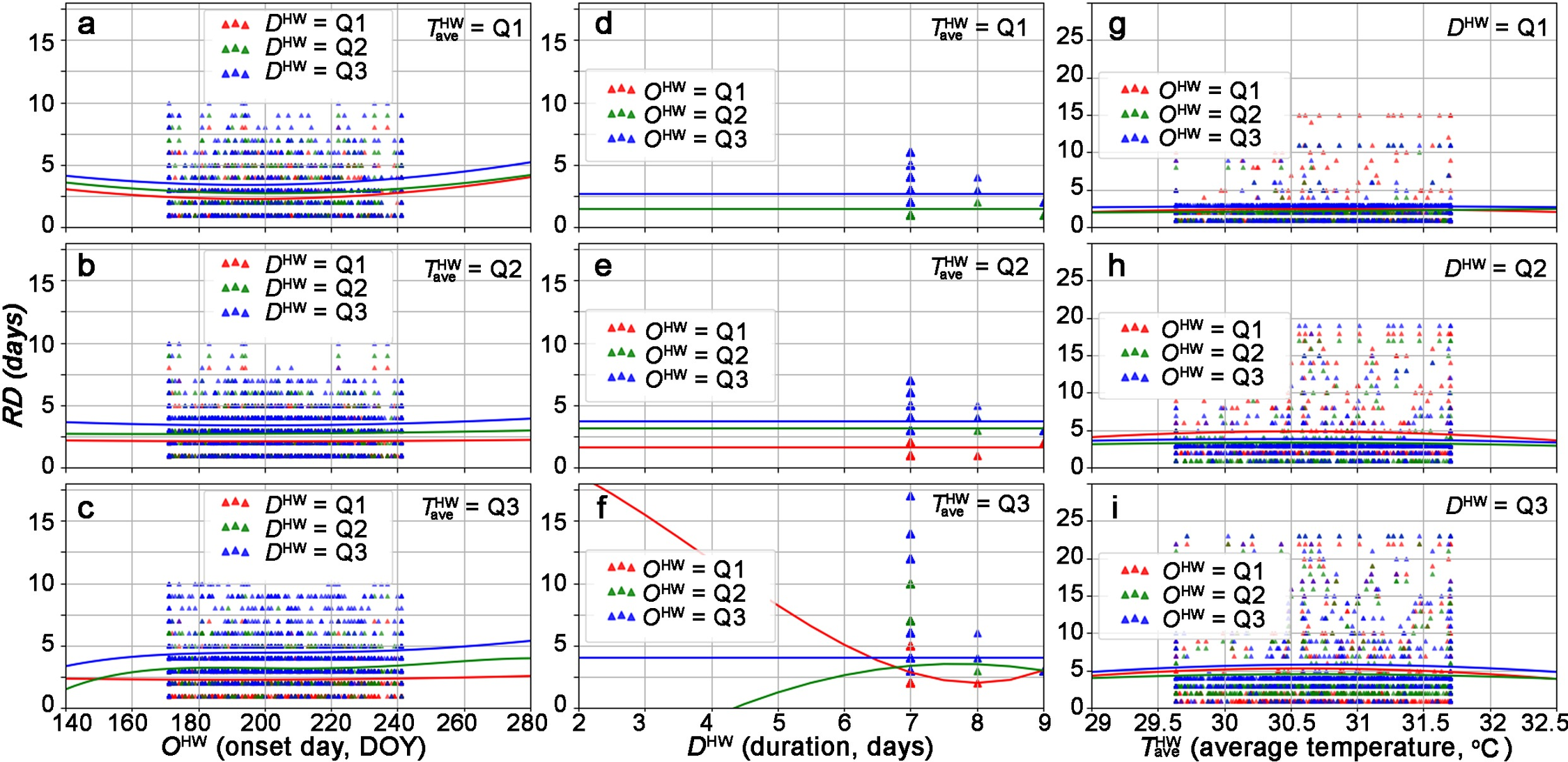

Supplement: S2 Fig — The relationships between RD and heat wave characteristics: (a-c) OHW, (d-f) DHW, and (g-i) TaveHW based on HW Definition III. Controlled variables are chosen as their first (Q1), second (Q2), and third quartile (Q3). (TIF) [file pntd.0007528.s006.tif]
